# Feasibility Randomised Control Trial of OptiMal: A Self-Management Intervention for Cancer Survivors

**DOI:** 10.3390/curroncol30120742

**Published:** 2023-11-29

**Authors:** Lauren Boland, Kathleen E. Bennett, Sinead Cuffe, Cliona Grant, M. John Kennedy, Deirdre Connolly

**Affiliations:** 1 Discipline of Occupational Therapy, Trinity College Dublin, D08 W9RT Dublin, Ireland; laboland@tcd.ie; 2 Data Science Centre, School of Population Health, RCSI University of Medicine and Health Sciences, D02 YN77 Dublin, Ireland; kathleenebennett@rcsi.ie; 3 Medical Oncologist, St James’s Hospital, James’ St, D08 W9RT Dublin, Ireland; scuffe@stjames.ie (S.C.); cgrant@stjames.ie (C.G.); jkennedy@stjames.ie (M.J.K.); 4 Trinity St James’s Cancer Institute, D08 W9RT Dublin, Ireland

**Keywords:** feasibility, physical and psychological health, self-management, cancer survivorship

## Abstract

Purpose: Cancer survivors can experience symptoms such as fatigue, pain and distress that persist for many months following treatment. These enduring symptoms often impact on participation in self-care activities, returning to school and/or work, and leisure and social activities. Self-management support is increasingly recognised as a core aspect of cancer survivorship care to reduce the impact of persistent symptoms. The purpose of this study was to examine the feasibility and potential effectiveness of a group-based self-management intervention, OptiMal, to improve the physical and psychological health of cancer survivors. OptiMal is a six-week intervention comprising weekly sessions on fatigue, stress and physical activity, diet and effective communication strategies. Methods: A feasibility randomised control trial was undertaken. Individuals up to two years after cancer treatment were randomised to OptiMal or usual care. Feasibility was examined through recruitment and retention metrics. Potential effectiveness was tested through patient-reported outcomes collected at baseline and three months post-intervention. Descriptive and inferential statistics were used to analyse study data. Results: Recruitment for this study was 32.5% (80/246 eligible individuals) with 77.5% retention at three-month follow-up (82.5% for intervention group and 72.5% for control group). Of those who attended the intervention, 19 (73%) attended all OptiMal sessions, indicating high adherence to the intervention. The majority of participants had breast cancer and were between 12 and 24 months post-treatment. The intervention group (n = 29) had statistically significant greater improvements in anxiety (*p* = 0.04) and health-related quality of life (health index score: *p* = 0.023, visual analogue score: *p* = 0.035) at three months post-intervention than the control group. Conclusions: Recruitment and retention in this study was similar to other cancer trials and the high adherence rate indicates that OptiMal is an acceptable self-management intervention for cancer survivors and warrants further investigation. OptiMal is intended to address symptoms reported across different cancer types. However, a limitation of this study was that the majority of participants had breast cancer, and therefore, generalisability of findings cannot be assumed for other cancer types. Future studies of OptiMal therefore need to use different strategies to recruit survivors of other cancer types.

## 1. Introduction

Cancer survival rates are increasing globally due to early diagnosis and effective cancer treatments [1]. However, cancer survivors experience persistent symptoms such as pain, fatigue, anxiety and depression post-treatment, which impact on their ability to return to pre-diagnosis roles and activities, thus affecting quality of life and wellbeing [2]. Self-management interventions are increasingly recognized as an integral aspect of cancer survivorship care in order to facilitate individuals to re-engage in pre-cancer roles and activities, and to improve quality of life [3]. Self-management is defined as an “individual’s ability to manage symptoms, treatment, physical and psychosocial consequences, and lifestyle changes inherent in living with a chronic condition” ([4] p. 178). It typically incorporates five core skills, namely problem solving, decision making, resource utilisation, effective communication and action planning. It distinguishes itself from traditional health education by its emphasis on the application of these skills to one’s own situation [5]. Self-management interventions assist cancer survivors to gain confidence to resume pre-cancer roles and routines by managing the impact of persistent symptoms. Although self-management is recommended for individuals to manage post-cancer treatment symptoms, many are unaware of how to achieve this [6].

Self-management interventions for cancer survivors address a wide range of commonly experienced post-treatment difficulties such as fatigue, reduced fitness, diet, anxiety, distress and cognitive impairments. Systematic reviews of self-management interventions report mixed findings on their outcomes. For example, Boland et al. [7] reported mixed outcomes for physical and psychosocial outcomes with limited evidence of the sustainability of effectiveness at long-term follow-up. In their systematic review of 41 studies, Cuthbert et al. [8] identified many limitations in the design of self-management interventions and in methodologies used to assess effectiveness of interventions. These reviews recommended that further research is required to rigorously evaluate effectiveness of self-management interventions for cancer survivors.

An occupational-therapy-led self-management intervention, OptiMal, which is based on principles of effective self-management, has been developed for people with multimorbidity [9,10]. OptiMal is a psychoeducational health behaviour change programme underpinned by self-management theory. The main objective of OptiMal is to develop participants’ knowledge and skills for managing physical and psychosocial symptoms to facilitate activity participation. The format of delivery is a combination of education by relevant healthcare professionals (depending on weekly topics), peer discussion and weekly goal setting. The self-management theory underpinning the content and delivery format of OptiMal is Bandura’s self-efficacy theory, which emphasizes the important influences of modelled and observed behaviour for changing health behaviours [11]. A core feature of OptiMal is providing opportunities for peer discussion, where participants learn from each other’s experiences and model self-management behaviours for long-term management of persistent physical and psychological symptoms post-treatment. Education is combined with goal setting where participants are facilitated to set realistic and achievable weekly goals to facilitate application of knowledge and skills into their daily routines, thus promoting sustainable self-management skills [5]. Goal setting is considered a key strategy in supporting individuals to develop meaningful and achievable self-management skills [12]. Goals are reviewed each week with the programme leader and all participants.

Due to the chronic nature of cancer-related symptoms, it is recommended that existing self-management interventions developed for individuals with chronic conditions be evaluated for cancer survivors [13]. Research to date demonstrates that OptiMal is effective in improving self-efficacy and health-related quality of life for individuals with multimorbidity [9,10]. Recent systematic reviews of self-management interventions for cancer survivors recommended further research to test the feasibility of existing self-management interventions for individuals with cancer [7,8]. These reviews specifically reported a lack of theory underpinning many interventions and a lack of strategies to support sustainable implementation of self-management knowledge and skills. The theoretical underpinning of OptiMal is self-efficacy aimed at increasing participants’ knowledge of and confidence to manage persistent symptoms following cancer treatment. Several self-efficacy strategies are embedded into the intervention including goal setting, goal review and self-regulation of barriers and facilitators for the implementation of self-management strategies. In addition to this, previous research on the effectiveness of self-management interventions reported a lack of sustainable change in health outcomes at long-term follow-up. However, studies of OptiMal have demonstrated sustained improvements in activity participation and mood at three months post-intervention. Given that OptiMal has potential to address some of the concerns and limitations raised in previous self-management intervention research, the aim therefore of this study was to examine the feasibility of OptiMal for individuals living with cancer.

## 2. Materials and Methods

### 2.1. Intervention

OptiMal is a multidisciplinary in-person group-based, six-week self-management intervention delivered once per week for 2.5 h. See Table 1 for further details of the weekly content and delivery format. OptiMal is facilitated by an occupational therapist, physiotherapist, and dietician. Each weekly session begins with education and group discussion on managing specific cancer-related symptoms. Topics include fatigue management; stress management; cancer-related cognitive impairments; exercise and physical activity (delivered by physiotherapist); diet and nutrition (delivered by dietician); and effective communication skills. Each session begins with one hour education on the weekly topic and includes peer discussion. During education sessions participants are encouraged to share self-management strategies that they have used previously. This is followed by a 30 min tea/coffee break to provide opportunities for participants to get to know each other, which facilitates open participation in peer discussion and sharing of self-management strategies. The final hour consists of goal setting and goal review, facilitated by the occupational therapist, to apply self-management strategies in daily activities during and after completion of the programme. Lorig et al. [5] recommended weekly goal setting and review to support participants to apply self-management skills and facilitate sustainability. All participants receive an OptiMal handbook that contains additional information and resources on each of the weekly topics, a copy of the slides presented each week and goal setting templates. To encourage weekly attendance at OptiMal, at the end of each week, the occupational therapist alerts participants to the topic that will be covered the following week and reminds participants of the importance of working on their goals over the week between each session.

OptiMal was delivered in the hospital in which the study was carried out. Four programmes were delivered during the study period by author LB who is an occupational therapist. Training in the content and delivery of OptiMal was provided to facilitators by author DC who designed OptiMal and delivered previous OptiMal training.

### 2.2. Study Design

A randomised control trial (RCT) was used to test the feasibility and examine the evidence for the potential effectiveness of OptiMal as a self-management intervention for cancer survivors guided by the CONSORT extension guidelines for feasibility and pilot studies [14]. The TIDieR checklist is included (Supplementary file) to describe the intervention. Ethical approval was received from the ethics committee of the recruiting hospital (approval number: 2014-12 Chairman’s Action (12). Informed consent was obtained from all individuals who participated in the study.

### 2.3. Study Population

Given that individuals with different types of cancer report difficulties with fatigue, distress and engaging in physical activity, individuals with any type of cancer were eligible for this study. Individuals over 18 years of age and between three months and two years after completing treatment (chemotherapy, surgery, radiation therapy) were eligible for inclusion. Individuals on long-term hormonal therapy were also eligible to participate. Exclusion criteria included individuals with communication or cognitive difficulties, clinical depression and/or anxiety, co-morbidities that would interfere with participation in the study (determined by the attending medical oncologist) and study participants who had not fully completed study measures at baseline and three-month follow-up. Billingham et al. [15] recommended a sample size of 36 people per arm for feasibility trials with a continuous endpoint. Therefore, to allow for 10% attrition, we aimed to recruit 80 individuals in total. Participants were recruited from outpatient oncology clinics of a national cancer centre.

### 2.4. Procedures

Members of the research team reviewed the medical charts of all individuals attending outpatient weekly oncology clinics. A participant information leaflet (PIL) was attached to the charts of those who met the inclusion criteria. When these individuals checked-in for their clinic appointment, they were provided with the PIL by the clinic administrator with responsibility for checking-in patients as they arrive for clinic appointments. Eligible participants then had time to read the PIL while waiting to see a member of the healthcare team. During this meeting, clinic staff (i.e., nurse and oncologists) followed up with patients on their interest in participating in the study. During this call, the researcher explained the intervention, the study processes, including randomisation, and the long-term commitment required from participants for completion of three-month follow-up questionnaires. Participants then attended a meeting with a member of the research team (LB). During this meeting, LB once again clarified the requirements for participating in the study, obtained informed consent (in writing or verbally based on participants’ preferences and abilities) and completed baseline study measures. Previous research of OptiMal recommended group sizes of six to eight participants. Therefore, when a total of 16 individuals agreed to participate in the study and had completed the baseline, they were assigned participant ID numbers. Following this process, randomisation was carried out by a biostatistician using a computer-generated randomisation sequence, which only the biostatistician (KB) had access to and therefore was concealed from those delivering the intervention [16]. The biostatistician was not involved in administering study questionnaires or intervention delivery. Those allocated to the intervention group received the six-week OptiMal intervention, which was delivered by a member of the research team (LB). Given the nature of the intervention, it was not possible to blind those delivering the intervention nor was it possible to blind participants to intervention assignment as those assigned to the intervention were required to attend the six-week OptiMal intervention. The control group received the usual care, which consisted of attending scheduled medical and outpatient appointments. Follow-up study measures were completed with a member of the research team three months following the intervention. As this was an unfunded study, LB completed the baseline and follow-up questionnaires with all study participants and delivered OptiMal to the intervention group and therefore was not a blinded assessor or interventionist. Following the three-month data follow-up, participants in the control group were provided with an OptiMal handbook and a relaxation CD.

### 2.5. Data Collection

#### 2.5.1. Primary Outcomes

As this was a feasibility study, the primary outcomes were: the number of patients screened for eligibility; the numbers who participated in the study (recruitment); the numbers of those who attended each week of the intervention as recorded by the occupational therapy facilitator (LB) (adherence); and the number of individuals who participated in the three-month follow-up (retention). Reasons for not participating in the study were also recorded. LB facilitated all four OptiMal programmes. Due to the nature of the intervention, it was not possible to blind LB to those allocated to the intervention. Training was provided to LB by DC who designed OptiMal. Fidelity of the intervention was monitored through a weekly log completed by LB. Acceptability of the intervention, examined through a qualitative methodology, is reported elsewhere [17].

#### 2.5.2. Secondary Outcomes

Many cancer survivors have difficulty resuming participation in daily activities and activities associated with life roles such as employment and parenting [2]. As one of the main objectives of OptiMal is to facilitate activity participation through symptom management, we used two activity-participation-related measures each testing two different aspects of activity participation. The Frenchay Activities Index, (FAI) [18], measures frequency of activity participation and the Canadian Occupational Performance Measure (COPM) [19] measures ability to perform, and satisfaction with, daily activities. The FAI consists of three subscales: domestic, leisure/work and outdoor activities [18]. Scores range from 0-45 with higher scores indicating greater activity engagement. The FAI has strong internal consistency (α = 0.83), criterion and construct validity and test–retest reliability (r = 0.96) [18]. Although this measure has not specifically been used with a cancer population, it is indicated for community-dwelling adults [20].

The Canadian Occupational Performance Measure (COPM) [19] measures satisfaction with the performance of daily activities related to self-care, work/education and leisure activities. Individuals rate their ability to perform and satisfaction with daily activities on a scale of 1 to 10 (1 = not able to do it/not at all satisfied and 10 = do it extremely well/extremely satisfied). Performance and satisfaction scores are summed (separately) and divided by the number of problem areas identified by the individual. A change of two points in performance and/or satisfaction levels is considered clinically significant [21]. The COPM is a valid and reliable outcome measure for use with community-dwelling individuals and has been used previously with cancer survivors [22].

The Functional Assessment of Chronic Illness Therapy (FACIT) measurement system is a collection of health-related quality of life questionnaires targeted to the management of chronic illness including cancer [23]. The FACIT—Fatigue (FACIT-F) short version is a 9-item scale within the FACIT measurement system. The FACIT-F measures physical and mental fatigue during a seven-day period from 0 (not at all) to 4 (very much) [24]. The total score is out of 52 with higher scores representing fewer symptoms of fatigue and higher quality of life. The FACIT-F is easy to use and has demonstrated reliability, validity and sensitivity to change [23]. The FACIT—fatigue scale demonstrates strong psychometric properties with internal consistency of 0.93–0.95 and test–retest reliability r = 0.87, and is able to discriminate between fatigue and mood (*p* < 0.001) [24].

The Hospital Anxiety and Depression Scale (HADS) is a reliable and valid 14-item self-assessment scale originally developed for use in a non-psychiatric outpatient setting [25]. It consists of two separate subscales for anxiety (HADS-A) and depression (HADS-D). Each item is rated on a 4-point scale (0 = not at all, 3 = yes definitely), with a maximum total score of 21 for each subscale, with higher scores indicating higher distress. HADS-A and HADS-D have both demonstrated strong internal consistency (α = 0.89 for HADS-A; α = 0.86 for HADS-D), good specificity and sensitivity, moderate intercorrelation and a distinct two-factor structure in general practice populations including women with breast cancer [26].

The Cognitive Failures Questionnaire (CFQ) assesses self-reported cognitive functioning in daily activities [27]. It consists of 25 items rated from 0-4 with the total score out of 100. Higher scores indicate higher levels of cognitive difficulties. This measure has demonstrated strong criterion validity, high inter-item consistency and test–retest reliability, and has been used with individuals with breast cancer [28]. This measure was included as cognitive impairments are frequently reported by cancer survivors and are exacerbated by physical and psychological symptoms [29].

The Stanford Chronic Disease Self-Efficacy Scale (SES) (6 item) [30] measures confidence levels in self-managing various elements of chronic diseases such as symptom control, role and emotional functioning, and communicating with health professionals [30]. Self-efficacy is considered a cornerstone of effective self-management [5]. The SES demonstrates good internal consistency (α = 0.91) and test–retest reliability (r = 0.72) in community-dwelling adults for a range of chronic conditions including cancer [31].

The EQ-5D-3L is a valid and reliable self-report questionnaire for assessing health-related quality of life (HRQOL [32]). It is a widely used measure consisting of a visual analogue scale and five dimensions of health and has strong test–retest reliability, inter-rater reliability, construct validity and discriminatory power across a number of chronic diseases [33]. The health states can be presented as the frequency of reported problems and converted into a single summary index [32].

## 3. Data Analysis

As the primary aim of this study was to examine feasibility metrics, descriptive statistics were used to measure recruitment, adherence and retention rates. Descriptive statistics were also used to explore participant demographics and clinical characteristics. Characteristics between those completing and not completing the three-month follow-up are also provided for comparison, although any statistical comparisons are not powered. The study was also not powered to detect significant differences between the intervention and control groups; however, in order to explore the potential effectiveness of the intervention, data from patient-reported outcome measures (PROMS) were examined for changes in scores using complete case analysis of all those who completed baseline and three-month follow-up questionnaires. Although this method of analysis has limitations, it is used in feasibility studies to explore the evidence for potential effectiveness [34]. Change scores in PROMS from baseline to three-month follow-up were calculated and the distribution of these change scores was examined for normality. For normally distributed scores, means and standard deviations (SDs) are presented, otherwise medians and interquartile ranges (IQRs) are presented. Statistical comparisons of normally distributed change scores between the two randomised groups were examined using two-way analysis of variance (ANOVA). The group by time interaction (intervention vs control = group effect; time = baseline to follow-up) was examined for differences between the groups. Data that were not normally distributed were examined using the Wilcoxon Rank Sum non-parametric test. Differences in demographic and clinical characteristics between the intervention and control groups at baseline were examined using the Chi-square test of independence. Differences between those who completed the study in both the intervention and control groups were also examined using the Chi-square test of independence. Effect sizes were calculated where data were normally distributed and interpreted according to procedures described by Cohen [35]: 0.2 to 0.50 = small to moderate; 0.51 to 0.80 = moderate to large; and >0.80 = large.

## 4. Results

### 4.1. Recruitment and Retention

Of 246 individuals identified through chart review as eligible to participate in the study, 80 individuals agreed to participate, giving a recruitment rate of 32.5%. Of the remaining 116 who were identified as eligible, it is unknown what proportion were invited and declined and what proportion were not invited as staff did not always have an opportunity to meet with all eligible individuals due to busy clinics. However, for individuals that declined to participate in the study, reasons included travel, work/family commitments, not in need of a self-management intervention and not interested.

**Figure 1:**
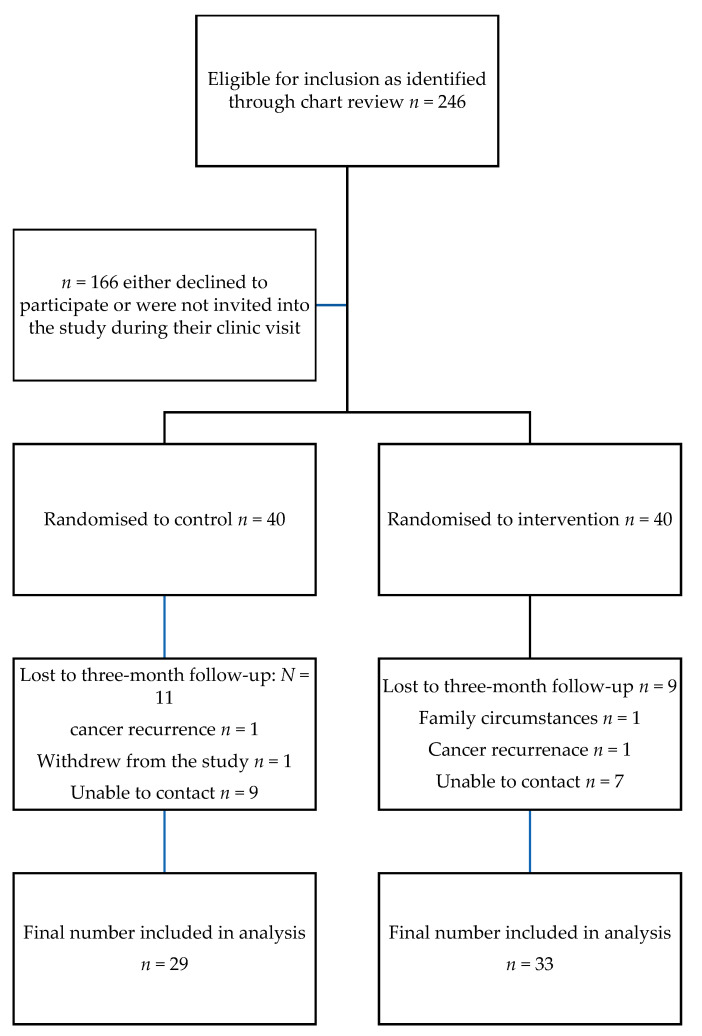
Recruitment and attrition.

All 80 who agreed to participate completed baseline assessments and were randomly assigned to the intervention (*n* = 40) or care as usual (*n* = 40). An overview of recruitment and retention is presented in Figure 1. Despite many efforts made to contact all study participants by both phone and email, eleven control group participants and seven intervention participants were lost to follow-up giving an overall retention rate of 77.5%.

### 4.2. Adherence to Intervention

Adherence to the intervention was measured through weekly attendance. Twenty-six of the forty individuals allocated to the intervention attended OptiMal. Of these 26 people, the majority (*n* = 19, 73%) attended all six sessions indicating a high adherence to, and acceptability of, the intervention. Reasons for non-attendance included medical appointments (*n* = 4), family responsibilities (*n* = 2) and personal reasons (*n* = 1).

### 4.3. Fidelity of Intervention Delivery

A fidelity logbook was completed weekly by LB and included any changes in content and/or delivery format. LB, who facilitated all four programmes during the study period, also attended the educational session on physical activity, which was delivered by a physiotherapist, and attended the nutrition sessions (delivered by a Dietician) to note modifications to the content and delivery format of OptiMal. The same physiotherapist and dietician delivered the physical activity and nutrition sessions in all four OptiMal programmes. No changes were identified in the content and/or delivery format during the study period.

The majority of participants were female (92.25%) and had breast cancer (72.5%). Other types of cancer included lymphoma, ovarian, head and neck, lung and oesophageal cancers. Table 2 presents the demographic and clinical characteristics of participants. The clinical and demographic characteristics were examined for differences between the intervention group and the control group at baseline. There were no significant differences between the two groups in these characteristics at baseline (Table 2).

A comparison was carried out on the baseline demographic and health characteristics between those who completed the study and those who were lost to follow-up for both the intervention and control groups. On examining differences between completers and non-completers in the intervention group, the only significant difference was the self-reported presence of chronic disease with 100% of non-completers reporting the presence of a chronic disease compared to 48.5% of completers (*p* ≤ 0.05). In the control group, the only significant difference between completers and non-completers was time since treatment, with 90.9% of non-completers having finished treatment within 12–24 month compared to 55.2% of completers (*p* < 0.05). See Supplementary Tables S1 and S2.

### 4.4. Comparison of Secondary Outcomes between Control and Intervention Participants

Although this study was not powered to detect significant differences in physical and psychological health outcomes, the measures were examined for indications of changes in these outcomes. Table 3 presents a comparison of the mean scores over time between the control and intervention participants for normally distributed outcome measures and effect sizes. Statistically significant improvements were observed from baseline to follow-up between the control and intervention groups for the HADS anxiety scale, (group by time interaction, *p* = 0.04), with a moderate effect size of 0.52. No significant group by time interactions were found for the other normally distributed measures.

Table 4 presents a comparison of the median scores over time between the control and intervention participants for non-normally distributed outcome measures. Statistically significant improvements were observed between the change scores of the control and intervention groups in the EQ-5D-3L QoL health states index score (*p* < 0.001) and the Euro QoL visual analogue scale (*p* = 0.035). The intervention group had a two-point increase in the COPM performance scale, which is considered a clinically important improvement [19] (Table 3). Although there were no statistically significant differences in the Cognitive Failure Questionnaire scores between the two groups, the scores of the intervention group improved, while the scores of the control group reduced, indicating increased cognitive difficulties for this group.

Table 5 presents data on the health state categories of the EQ-5D-3L. The proportion of intervention participants reporting “no problems” increased in four of the five categories, with the highest proportion reporting improvements in “usual activities”. This is in contrast to the control group where reported difficulties in the five categories overall stayed the same, indicating little change over the three-month follow-up period.

## 5. Discussion

Cancer survivors report that persistent physical and psychological symptoms post-cancer treatment interfere with their ability to re-engage in valued roles and activities. The need for self-management interventions to provide individuals with knowledge and skills to manage the impact of persistent symptoms has been identified. The aim of this study therefore was to test the feasibility of a pre-existing self-management intervention, OptiMal, for individuals following the completion of cancer treatment. The study demonstrated comparable recruitment rates to other cancer-related trials, high adherence to the intervention and low attrition rates. Following attendance at OptiMal, there were statistically significant improvements for the intervention participants for anxiety and quality of life. Other outcome measures showed greater improvements for the intervention participants but did not reach significance.

### Feasibility

The recruitment rate for this study was 32.5%. A recent systematic review of recruitment rates in cancer-related trials identified a median recruitment rate of 38% and cited similar reasons as identified in this current study for lack of participation, such as difficulty with travel and lack of interest [36]. The recruitment strategy for this current study involved clinic staff providing individuals with the study information leaflet. For ethical reasons, the research team was not permitted to directly approach eligible participants to inform them of the study. Discussing the commitment required to participate in a study and providing opportunities for potential participants to discuss expectations for participation is considered important for study recruitment [37]. For future OptiMal studies, ethical permission will be sought to mail study information leaflets to potential participants with a follow-up phone call by a member of the study team.

The retention rate in this study (77.5%) is considered acceptable for an RCT with a three-month follow-up. The loss of study participants to follow-up can introduce bias and impact on the generalisability, validity and reliability of study findings [38]. Apart from offering assistance in completing follow-up questionnaires, no other retention strategies were used in the study. Future studies of OptiMal could utilise additional retention strategies such as regular contact with study participants during the follow-up phase and, if possible, provide a monetary incentive [37].

The majority of study participants were women with breast cancer. Breast cancer is one of the most prevalent cancer types internationally and accounts for 29% of all cancer types in Ireland [1]. A recent systematic review of self-management interventions identified breast cancer survivors as the majority participants [7]. Van de Poll-Franse et al. [39] also reported that breast cancer survivors utilised healthcare services more frequently than other cancer survivors. This therefore limits the generalisability of the findings from this study for other cancer types. The rationale for including all cancer types in the current study was that the focus of the intervention is on managing common symptoms across all cancer types including fatigue, distress and difficulty resuming physical activities. However, based on the findings of this study, it is possible that self-management interventions for cancer survivors may need to be customised for different cancer types to address cancer-specific difficulties. Participants of a recent study of OptiMal for individuals with oesophageal cancer identified the benefit of meeting others with the same cancer type and sharing cancer-specific self-management strategies [40].

In total, seven men participated in the current study. The low uptake of self-management programmes by men was highlighted by Galdas et al. [41] who reported that men are reluctant to seek help to manage long-term health difficulties due to it being perceived as an inability to cope with the impact of their cancer. However, men can also experience a range of post-treatment health difficulties, and therefore, self-management interventions are recommended [41]. Targeted recruitment strategies such as advertising the study in men’s cancer support groups may increase male recruitment.

A high attendance rate by the intervention group indicates that OptiMal was an acceptable intervention with 73% of the intervention group attending all six sessions of OptiMal. Weekly attendance involved considerable commitment from participants as they were required to attend for 2.5 h/week in addition to time required for travelling to and from the intervention centre. Participants were also required to work on weekly goals related to implementation of self-management strategies between weekly sessions. However, the high levels of attendance would indicate that this time commitment was not considered problematic by intervention participants. Qualitative interviews completed as part of this current study supported this finding with intervention participants reporting high acceptability of the content, intervention format, duration and time commitment [17].

Demographic and clinical characteristics were compared for those who completed the study and those who were lost to follow-up in both the intervention and control group participants. The only significant difference between completers and non-completers in the intervention group was the self-reported presence of chronic conditions for all non-completers (100%). Chronic conditions present a wide range of disease-specific symptoms such as nausea, pain, fatigue and distress that may exacerbate cancer-related symptoms. This finding indicates that future studies of OptiMal should screen for the impact of chronic conditions, including symptom severity, and include an assessment of the impact of disease-specific symptoms on participants’ physical and psychological health. Assessment findings could then be used to tailor the content and delivery format of OptiMal to accommodate individuals with chronic conditions. The presence of chronic conditions may also impact on ability to participate in longitudinal studies, and therefore, the study team may also need to provide additional supports to retain study participants with chronic conditions.

For the control group, the only significant difference between completers and non-completers was that a higher proportion of the non-completers were between 12 and 24 months since completion of their treatment. Perhaps, given the time since completing treatment, these individuals had resumed pre-diagnosis activities and roles such as returning to work and therefore could not prioritise participation in the study over these activities. This further supports the need to tailor intervention delivery and format and employ a range of strategies to retain participants in intervention trials. Such strategies include flexibility in intervention delivery, regular communication from the study team and reimbursement for travel and time [42].

There were no other significant differences in the demographic characteristics of age, marital status, employment and education levels for completers and non-completers in both arms. This is in contrast with previous research that reported these variables can influence participation in self-management interventions trials [36]. Although not significantly different, a higher proportion of non-completers in the intervention group were living with family members. Perhaps these individuals did not feel the need to continue with the intervention as they were receiving adequate support from family members. Previous research identified the impact of family support for developing survivorship management strategies [43].

## 6. Potential Effectiveness

A secondary aim of this study was to examine changes in physical and psychological outcome measures as an indicator of the potential effectiveness of OptiMal in improving the physical and psychological health of cancer survivors. The results indicate that participation in OptiMal significantly improved psychological health and quality of life three months following completion of the intervention. Although participation in daily activities showed greater improvement for the intervention participants, there was no statistically significant difference between the two groups. However, as this was a feasibility study, the sample size was not powered to test for statistical significance.

Anxiety and distress are reported as frequent outcomes of a cancer diagnosis and cancer treatment [44]. Previous studies indicated that self-management interventions may be beneficial for reducing anxiety and depression [2,44]. In this current study, there were significant differences between the control and intervention groups in anxiety levels at three-month follow-up. A recent systematic review of self-management interventions for cancer survivors reported significant improvements in anxiety immediately following self-management interventions; however, these improvements were not sustained at follow-up [7]. The significant improvements at three-month follow-up in this current study would suggest that OptiMal has potential for a sustained impact in terms of reducing anxiety for cancer survivors. However, this needs further investigation in a definitive intervention trial.

A study of HRQOL in long-term cancer survivors reported that reduced quality of life can persist for up to 10 years following cancer treatment [45]. In this current study, there were significant differences in HRQOL in favour of the intervention participants at three-months post-intervention with a median change score of 0.07. Changes of between 0.07–0.12 for the EQ-5D index score have been identified as clinically important for cancer survivors [46].

Although the intervention group had greater improvements in participation in daily activity, this difference was not statistically significant. Based on baseline FAI scores, both groups appeared to have relatively high levels of activity participation at the start of the study, and therefore, the lack of a significant improvement could be related to the timing of intervention delivery. The majority of participants in the current study were one-year post-cancer treatment and may have already returned to pre-treatment activity levels prior to participating in OptiMal. Fleischer and Howell [47] interviewed breast cancer survivors six months post-treatment and reported that many participants had learned to manage post-treatment symptoms, which interfered with activity participation, by this time. The most appropriate time for delivering self-management interventions to cancer survivors has not yet been determined, but perhaps it is more useful closer to the completion of treatment. Future self-management studies could examine this further in order to identify the most suitable time to provide self-management interventions to support activity participation.

Fatigue is a persistent symptom for cancer survivors which impacts considerably on activity participation [2]. Our feasibility study showed an effect size between the intervention and control groups of 0.27 for the FACIT-F score between baseline and three-month follow-up, which is considered a small to moderate effect. Two recent systematic reviews of self-management interventions reported a lack of long-term effectiveness for cancer-related fatigue [7,8]. The causes of, and strategies for, managing fatigue are one of the weekly topics of OptiMal. Perhaps this is not sufficient intervention for a symptom that impacts significantly on physical and psychological health [2]. A recent review of exercise interventions in the management of cancer-related fatigue suggested that intervention dose and duration impacted on fatigue outcomes [48]. Therefore, although OptiMal includes strategies for managing fatigue, perhaps a focused fatigue-specific intervention is required to facilitate significant improvements in fatigue. Other secondary outcomes, namely anxiety, depression, self-efficacy and satisfaction with performance of daily activities suggested greater improvements for the intervention group; however, a definitive intervention trial is required to examine the effectiveness of OptiMal on these outcomes.

## 7. Conclusions

Cancer survivors experience persistent symptoms post-treatment that impact on activity participation and quality of life. It is recommended that existing self-management interventions are examined for their effectiveness in improving the physical and psychological health of cancer survivors. The findings of this study suggest that OptiMal is a feasible and acceptable self-management intervention for cancer survivors with potential for improvements in anxiety and health-related quality of life. A definitive intervention trial is required to establish effectiveness.

## Figures and Tables

**Table 1:** Weekly content and MDT facilitators of OptiMal.

Weekly Session 2.5 h/Session	Content	Facilitator/s
Week 1	Introduction to OptiMal Principles of self-management Introduction to SMART goal setting	Occupational Therapist
Week 2	Cancer-Related Fatigue (CRF) Causes of CRF and factors that exacerbate CRF Fatigue management strategies Weekly goal setting	Occupational Therapist
Week 3	Exercise and Physical Activity Role of physical activity in recovery from cancer treatment Benefits of physical activity Recommendations for physical activity in cancer survivorship Weekly goal setting and review	Physiotherapist and Occupational Therapist
Week 4	Mental Health and Cancer-related Cognitive Impairments Factors that impact on mental health Strategies for optimising mental health Cancer-related cognitive impairments Cognitive-based strategies for management of cancer-related cognitive impairments Weekly goal setting and review	Occupational Therapist
Week 5	Healthy Eating Benefits of balanced diet post-treatment Integrating healthy eating habits into daily routines Weekly goal setting and review	Dietician and Occupational Therapist
Week 6	Effective Communication Barriers to, and facilitators of, communicating with health professionals, employers and family members Weekly goal review	Occupational Therapist

**Table 2:** Participant characteristics at randomisation.

	Control Group (*n* = 40)	Intervention Group (*n* = 40)	*p*-Value
**Mean Age (SD)**	50.4 (11.75)	51.68 (11.73)	0.48
**Gender**			0.69
Male *n* (%)	3 (7.5%)	4 (10%)	
Female *n* (%)	37 (92.5%)	36 (90%)	
**Type of Cancer**			0.61
Breast *n* (%)	30 (75%)	28 (70%)	
Other *n* (%)	10 (25%)	12 (30%)	
**Type of Treatment**			0.98
Surgery, Chemotherapy and Radiation Therapy *n* (%)	21 (52.5%)	20 (50%)	
Other *n* (%)	19 (47.5%)	20 (50%)	
**Time since treatment completion**			0.96
<12 months: *n* (%)	14 (35%)	12 (30%)	
12–24 months: *n* (%)	26 (65%)	28 (70%)	
**Marital Status**			0.36
Married *n* (%)	20 (50%)	24 (60%)	
Other *n* (%)	20 (50%)	16 (40%)	
**Living Situation**			0.49
Family *n* (%)	34 (85%)	36 (90%)	
Other *n* (%)	6 (15%)	4 (10%)	
**Level of Education**			0.49
Primary-Leaving Cert *n* (%)	21 (52.5%)	24 (60%)	
College/University *n* (%)	19 (47.5%)	16 (40%)	
**Chronic Condition (Self-reported)**			0.17
Yes *n* (%)	13 (32.5%)	19 (47.5%)	
No *n* (%)	27 (67.5%)	21 (52.5%)	
**Employment Status Prior to Treatment**			0.63
Full-time *n* (%)	18 (45%)	18 (45%)	
Part-time *n* (%)	6 (15%)	9 (22.5%)	
Other (*n*, %)	16 (40%)	13 (32.5%)	
**Employment Status After Treatment**			0.17
Full-time *n* (%)	10 (25%)	9 (22.5%)	
Part-time *n* (%)	6 (15%)	13 (32.5%)	
Other *n* (%)	24 (60%)	8 (20%)	

**Table 3:** Comparison of mean scores over time (baseline and follow-up) between the groups for normally distributed outcome measures and effect size.

	Control Group (*n* = 29)			Intervention Group (*n* = 33)				
Measures (Score Range)	Baseline Mean (SD)	Three-Month Follow-Up Mean (SD)	Mean Change (SD)	Baseline Mean (SD)	Three-Month Follow-Up Mean (SD)	Mean Change (SD)	Effect Size	(t-Statistic) *p*-Value
**FAI ^1^** **(0–45)**	33.3 (5.94)	35.3 (3.44)	2.07 (4.0)	34.1 (5.46)	35.9 (4.33)	1.97 (3.6)	−0.06	(0.99) 0.89
**HADS-A ^2^** **(0–21)**	7.66 (4.58)	8.00 (4.80)	0.34 (2.7)	9.21 (3.70)	7.76 (3.23)	−1.45 (3.9)	−0.52	(2.05) * **0.04**
**HADS-D ^3^** **(0–21)**	5.34 (3.50)	5.31 (4.18)	−0.07 (3.04)	4.97 (3.21)	4.48 (3.33)	−0.49 (3.7)	−0.12	(0.47) 0.61
**SES ^4^** **(1–10)**	7.08 (1.54)	7.38 (1.62)	0.30 (1.2)	6.93 (1.71)	7.75 (1.85)	0.87 (1.9)	0.24	(−1.37) 0.22
**FACIT-F ^5^** **(0–52)**	31.72 (12.75)	32.14 (11.59)	0.48 (6.4)	33.18 (10.71)	35.67 (10.44)	2.48 (8.3)	0.27	(−1.05) 0.28
**COPM-S ^6^** **(1–10)**	3.52 (2.77)	5.69 (2.43)	2.2 (2.6)	3.76 (2.12)	6.36 (2.32)	2.6 (2.2)	0.17	(−0.64) 0.52

^1^ Frenchay Activities Index; ^2^ Hospital Anxiety Depression Scale—Anxiety; ^3^ Hospital Anxiety Depression Scale—Depression; ^4^ Stanford Self-Efficacy Scale; ^5^ Functional Assessment of Chronic Illness Therapy—Fatigue; ^6^ Canadian Occupational Performance Measure—Satisfaction, * Significant *p*-values.

**Table 4:** Comparison of median (interquartile (IQR) range) scores over time (baseline and follow-up) between the groups for non-normally distributed outcome measures.

	Control Group (*n* = 29)			Intervention Group (*n* = 33)			
Measures (Score Range)	Baseline Median (IQR)	Three-Month Follow-Up Median (IQR)	Median (IQR) Change Score	Baseline Median (IQR)	Three-Month Median (IQR)	Median (IQR) Change Score	(z-Score) *p*-Value
**EQ-5D-3L Health Index score (0–1)**	0.73 (0.1)	0.73 (0.2)	0 (−0.2)	0.68 (0.1)	0.75 (0.2)	0.07 (−0.20)	(−3.3) 0.001 *
**EQ-5D-3L VAS**	67.5 (31.25)	70 (30)	0 (−5.00)	70 (12.5)	75 (15)	4.18 (14.9)	(−2.1) 0.035 *
**COPM-P ^1^** **(1–10)**	5 (2.6)	6 (2.4)	1.2 (3.0)	5 (1.5)	7 (2.4)	2 (2.3)	(−0.42) 0.41
**CFQ ^2^** **(0–100)**	34 (22)	39 (24)	1 (7.5)	40 (28)	39 (24)	−1.0 (1.0)	(−1.1) 0.26

^1^ Canadian Occupational Performance Measure—Performance; ^2^ Cognitive Failures Questionnaire, * Significant *p*-values.

**Table 5:** Euro QoL Health States (EQ-5D-3L) of control and intervention groups at baseline and three-month follow-up.

EQ-5D Outcomes	Control Group		Intervention Group	
	Baseline (*n* = 29) (%)	3-month follow-up (*n* = 29) (%)	Baseline (*n* = 33) (%)	3-month follow-up (*n* = 33) (%)
**Mobility**				
No Problems	23 (79.3%)	17 (58.6%)	20 (60.6%)	24 (72.7%)
Moderate/Severe Problems	6 (20.7%)	12 (41.4%)	13 (29.4%)	9 (27.3%)
**Self-Care**				
No Problems	29 (100%)	29 (100%)	33 (100%)	33 (100%)
**Usual Activities**				
No Problems	15 (51.7%)	18 (62.1%)	14 (42.4%)	21 (63.6%)
Moderate/Severe Problems	14 (48.3%)	11 (37.9%)	19 (57.6%)	12 (36.4%)
**Pain/Discomfort**				
No Problems	9 (31%)	9 (31%)	7 (21.2%)	13 (39.4%)
Moderate/Severe Problems	20 (69%)	20 (69%)	26 (78.8%)	20 (60.6%)
**Anxiety/Depression**				
No Problems	14 (48.3%)	14 (48.3%)	7 (21.2%)	13 (39.4%)
Moderate/Severe Problems	15 (51.7%)	15 (51.7%)	26 (78.8%)	20 (60.6%)

## Data Availability

Data that support the findings of this study are available from the corresponding author upon reasonable request.

## Supplementary Materials

The following supporting information can be downloaded at: https://www.mdpi.com/article/10.3390/curroncol30120742/s1, Table S1: Comparison of Participant characteristics in control arm: Completers vs non-completers at three month follow-up; Table S2: Comparison of Participant characteristics in intervention arm: Complete vs not complete at three month follow-up.
