# Supplementary material for: Feasibility Randomised Control Trial of OptiMal: A Self-Management Intervention for Cancer Survivors
Source: Curr Oncol. 2023 Nov 29;30(12):10195–210. doi: 10.3390/curroncol30120742 (PMC10742444; doi:10.3390/curroncol30120742)
Supplement: Supplementary file 1 [file curroncol-30-00742-s001.zip › curroncol-2705593-supplementary.pdf]

Table S1 Comparison of Participant characteristics in control arm: Completers vs non-completers at three month follow-up

	Complete (n=29)	Not complete (n=11)
**Mean Age (SD)**	52.1 (12.4)	46 (9.0)
**Gender**		
Male n (%)	2 (6.9%)	1 (9.1%)
Female n (%)	27 (93.1%)	10 (90.9%)
**Type of Cancer**		
Breast n (%)	20 (75%)	8 (72.7%)
Other n (%)	9 (25%)	3 (27.3%)
**Type of Treatment**		
Surgery, Chemotherapy and Radiation Therapy n (%)	13 (44.8 %)	8 (72.7%)
Other n (%)	16 (55.2%)	3 (27.3%)
**Time since treatment completion**		
<12 months: n (%)	13 (44.8%)	1 (9.1%) *
12 – 24 months: n (%)	16 (55.2%)	10 (90.9%)
**Marital Status**		
Married n (%)	14 (48.3%)	6 (54.6%)
Other n (%)	15 (51.7%)	5 (45.4%)
**Living Situation**		
Family n (%)	25 (86.2%)	9 (81.8%)
Other n (%)	4 (13.8%)	2 (18.2%)
**Level of Education**		
Primary-Leaving Cert n (%)	15 (51.7%)	6 (54.6%)
College/University n (%)	14 (48.3%)	5 (45.4%)
**Chronic Condition (Self-reported)**		
Yes n (%)	11 (62.1%)	9 (81.8%)
No n (%)	18 (37.9%)	2 (18.2%)
**Employment Status Prior to Treatment**		
Full-time n (%)	20 (69.0%)	6 (54.5%)
Part-time n (%)	3 (10.3%)	3 (27.3%)

Other (n, %)	6 (20.7%)	2 (18.2%)
**Employment Status After Treatment**		
Full-time n (%)	15 (51.7%)	4 (36.4%)
Part-time n (%)	4 (13.8%)	2 (18.2%)
Other n (%)	10 (34.5%)	5 (45.4%)

Table S2: Comparison of Participant characteristics in intervention arm: Complete vs not complete at three month follow-up

	Complete (n=33)	Not complete (n=7)
**Mean Age (SD)**	52.5 (10.7)	47.71 (6.16)
**Gender**		
Male n (%)	4 (12.1%)	0 (0%)
Female n (%)	29 (87.9%)	7 (100%)
**Type of Cancer**		
Breast n (%)	23 (70%)	5 (65%)
Other n (%)	11 (30%)	2 (35%)
**Type of Treatment**		
Surgery, Chemotherapy and Radiation Therapy n (%)	15 (45.4%)	5 (71.4%)
Other n (%)	18 (54.6%)	2 (28.6%)
**Time since treatment completion**		
<12 months: n (%)	10 (30.3%)	2 (28.6%)
12 – 24 months: n (%)	23 (69.7%)	5 (71.4%)
**Marital Status**		
Married n (%)	21 (63.6%)	3 (42.9%)
Other n (%)	12 (36.4%)	4 (57.1%)
**Living Situation**		
Family n (%)	29 (87.9%)	7 (100%)
Other n (%)	4 (12.1%)	0 (0%)
**Level of Education**		
Primary-Leaving Cert n (%)	14 (42.4%)	5 (71.4%)
College/University n (%)	19 (47.6%)	2 (28.6%)
**Chronic Condition (Self-reported)**		
Yes n (%)	16 (48.5%)	7 (100%) *
No n (%)	17 (51.5%)	0 (0%)
**Employment Status Prior to Treatment**		
Full-time n (%)	20 (60.6%)	2 (28.6%)
Part-time n (%)	7 (21.2%)	2 (28.6%)
Other (n, %)	6 (18.2%)	3 (42.9%)

**Employment Status After Treatment**		
Full-time n (%)	11 (33.3%)	1 (14.3%)
Part-time n (%)	9 (27.3%)	4 (57.1%)
Other n (%)	13 (39.4%)	2 (28.6%)
